# Cocaine Detection by a Laser-Induced Immunofluorometric Biosensor

**DOI:** 10.3390/bios11090313

**Published:** 2021-09-03

**Authors:** Martin Paul, Robert Tannenberg, Georg Tscheuschner, Marco Ponader, Michael G. Weller

**Affiliations:** Division 1.5 Protein Analysis, Federal Institute for Materials Research and Testing (BAM), Richard-Willstätter-Strasse 11, 12489 Berlin, Germany; martin.paul@bam.de (M.P.); robert.tannenberg@bam.de (R.T.); georg.tscheuschner@bam.de (G.T.); marco.ponader@bam.de (M.P.)

**Keywords:** online detection, security, flow injection assay, monoclonal antibody, fluorescence microscope, microfluidic systems, monolithic column, laser-induced fluorescence detector (LIF), low-cost, high-speed

## Abstract

The trafficking of illegal drugs by criminal networks at borders, harbors, or airports is an increasing issue for public health as these routes ensure the main supply of illegal drugs. The prevention of drug smuggling, including the installation of scanners and other analytical devices to detect small traces of drugs within a reasonable time frame, remains a challenge. The presented immunosensor is based on a monolithic affinity column with a large excess of immobilized hapten, which traps fluorescently labeled antibodies as long as the analyte cocaine is absent. In the presence of the drug, some binding sites of the antibody will be blocked, which leads to an immediate breakthrough of the labeled protein, detectable by highly sensitive laser-induced fluorescence with the help of a Peltier-cooled complementary metal-oxide-semiconductor (CMOS) camera. Liquid handling is performed with high-precision syringe pumps and microfluidic chip-based mixing devices and flow cells. The biosensor achieved limits of detection of 7 ppt (23 pM) of cocaine with a response time of 90 s and a total assay time below 3 min. With surface wipe sampling, the biosensor was able to detect 300 pg of cocaine. This immunosensor belongs to the most sensitive and fastest detectors for cocaine and offers near-continuous analyte measurement.

## 1. Introduction

In the recent European Drug Report 2021 [1], it was stated that the market for cocaine in Europe is still growing. Current data show that both the number of seizures and their volumes are at a historic high. A total of 213 tons of cocaine were seized in 2019 from the EU member states, with an estimated value of more than EUR 11 billion. One of the main tasks of police and customs authorities at harbors, airports, and borders is the control of illegal drug trafficking. Many different methods, sometimes of high sophistication, have been tried and used to transport and distribute illegal drugs. Despite the enormous efforts to reduce the import of illegal drugs, these activities cannot be very effective. The commonly used Scott’s color test seems to show poor specificity, which leads to false negative or false positive results [2]. Also, the presence of masking substances effectively hinders the detection of cocaine by color tests and mid-infrared analysis (MIR) [3]. This situation puts some pressure on the development of improved methods for drug detection; a comprehensive review was recently published by Interpol [4]. Today, drug-sniffing dogs seem to be the gold standard for the search for this purpose. It is obvious that this method is quite expensive and is limited to specific areas of high drug trafficking activity. In between, normal police officers also use immunochromatographic wipe tests for surfaces and other quick tests, e.g., for saliva or hair for the testing of suspects. In addition, mobile devices, such as ion mobility spectrometers (IMS), are available. Nevertheless, their applicability seems to be limited [5], and more powerful methods may be desirable. Several excellent reviews have been published to give a broad insight into conventional and emerging techniques for the detection of illegal drugs and their metabolites. Reviews or other papers covering the detection of drugs, e.g., via the testing of hair [6,7,8,9], the use of molecularly imprinted polymers [10,11], aptamers [12], ion mobility spectrometry [10,11], mass spectrometry [13,14,15,16,17], hybrid methods [18], electrochemical methods [19,20], biosensors [12,21,22,23,24,25,26,27,28,29,30,31,32,33,34], and immunoassays [35,36,37] have been published. Quite popular in analytical chemistry was the detection of cocaine on banknotes [38,39,40,41,42,43,44,45]. It can be concluded that cocaine detection is still an active field of research and development.

Limitations of existing approaches comprise the cost and mobility of the systems, speed, sensitivity, and particularly, the selectivity of the sensors. “False negatives” seem to be frequent if the amounts of the distributed cocaine are compared with the seized amounts. False positives can have quite unpleasant effects for the falsely suspected and the workflows at the security checks at airports and other critical traffic junctions.

In this work, a laser-induced immunofluorometric biosensor for cocaine is presented, which shows quite a few specific advantages. First of all, this sensor displays an exquisite selectivity, which is based on the use of proven monoclonal antibodies of high specificity. Antibody IP3G2 was frequently applied in previous studies and showed only minor cross-reactivities limited to closely related substances [21,42].

**Figure 1:**
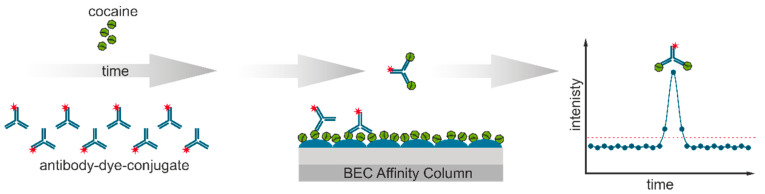
Assay principle: Fluorescently labeled anti-cocaine antibody is mixed with the sample and injected onto an affinity column coated with a conjugate based on the cocaine derivative benzoylecgonine (BEC). Unbound antibodies are immediately detected by an increased fluorescence signal.

A special benefit of the format presented here is the combination of an antibody-excess regime with a competitive assay, which is quite uncommon. This setup can overcome an affinity limitation of conventional competitive immunoassays, which are the standard format for small molecule assays. Hence, laser-induced immunofluorometric biosensors can achieve very low limits of detection in combination with short response times. However, it could be objected that the continuous flow of labeled antibodies might lead to excessive consumption of reagents. It turned out that—mainly due to the extremely sensitive detection—this is not the case. This also means that usually, not even a regeneration of the affinity column is necessary. Previously, high sensitivity detection by laser-induced immunofluorometric biosensors was demonstrated for the high explosive TNT [46] in aqueous samples. However, some external incubation steps were still required, resulting in longer measurement times and a discontinuous baseline. In this work, previous limitations were overcome using microfluidics, allowing for continuous mixing and incubation, resulting in online measurements resulting in much faster detection with convenient sample introduction. Also, intensity determination and data evaluation were improved to allow for an easier and more robust signal interpretation. Additionally, besides detection in aqueous solutions only, a surface sampling method suitable for this detector was developed, showing very high sensitivity for the analyte cocaine in a surface testing setup. The application of the method (see Figure 1) to the polar tropane alkaloid cocaine shows broad applicability of the immunofluorometric biosensor combined with excellent sensitivity. In this biosensor, a proven antibody against cocaine or benzoylecgonine, respectively, was used (clone IP3G2). According to the datasheet of the monoclonal antibody, the immunogen was a benzoylecgonine-KLH conjugate; the affinity constant is given as 5.8∙10^9^ L/mol.

## 2. Materials and Methods

### 2.1. Reagents, Buffers, Materials, and Equipment

White, flat-bottom high binding 96-well microtiter plates (655074) were acquired from Greiner Bio-One (Frickenhausen, Germany), PD SpinTrap G-25 were obtained from Cytiva (Washington, DC, USA), monoclonal anti-benzoylecgonine/cocaine (BEC) antibody IP3G2 (mouse, subtype IgG_1_) was obtained from Genway (San Diego, CA, USA), cross-reactivities are given in [21]. Goat anti-mouse horseradish peroxidase (HRP)-conjugated antibody (115-035-146) was obtained from Jackson immune research (Cambridge, UK), fluorescence dye Dy-654-NHS was purchased from Dyomics (Jena, Germany). According to the manufacturer, the following properties of the fluorescent dye Dy-654 are given: excitation/emission max. 653/677 nm (in ethanol), molar absorbance: 220.000 M^−1^cm^−1^, soluble in water, methanol, and DMF (https://dyomics.com/en/products/red-excitation/dy-654, accessed on 1 September 2021). Bovine serum albumin (BSA) >98% (A7906), diethoxy(3-glycidyloxypropyl)-methyl silane (539252), ProClin300 (8912-U) were purchased from Sigma-Aldrich (Taufkirchen, Germany). Hydrochloric acid (HCl, 84415) was purchased from Fluka, and cyano-4-hydroxycinnamic acid (CHCA) was bought from Bruker Daltonics (Bremen, Germany), sodium bicarbonate (1940) and potassium hydroxide (121515) were obtained from AppliChem (Darmstadt, Germany). Chemiluminescent Substrate (SuperSignal West Atto Ultimate Sensitivity, A38555) was bought from Thermo Scientific (Waltham, USA), Tween 20 (37470.01) was bought from Serva (Heidelberg, Germany), absolute ethanol (2246) from Th. Geyer (Renningen, Germany) and labeling grade DMF (13050) was bought from Lumiprobe (Hannover, Germany). Cocaine hydrochloride (Extra pure, Cat. No. 1.02562.0005) from Merck (Darmstadt, Germany) and benzoylecgonine tetrahydrate (D745) from National Measurement Institute of Australia, North Ryde) were kindly supplied by BAM Division 1.8. Vitrapor5 glass monoliths were acquired from Robu (Hattert, Germany), and ultrapure water (MilliQ) was supplied by a Milli-Q Synthesis A10 system (Merck, Germany). Cotton swabs Cien, EAN 2047 6830 were acquired from Lidl (Neckarsulm, Germany). The optical system is described elsewhere [46]. The microfluidic flow cell (10000091), the microfluidic micromixer (10000759), and the TOPAS (10000443) substrate were acquired from Microfluidic ChipShop (Jena, Germany), the injection valve (5067–4158) from Agilent (Santa Clara, CA, USA), and a Fusion 4000 syringe pump was acquired from Chemyx (Stafford, TX, USA). Matrix-Assisted Laser Desorption/Ionization Time-of-Flight (MALDI-TOF) mass spectrometry was performed on a Bruker Autoflex Max MS, and chemiluminescence was measured with a Synergy H1 spectrometer from Biotek (Winooski, VT, USA). Data evaluation was performed with Python 3.7 in Anaconda (Austin, TX, USA) and Origin 2018G (Northampton, MA, USA).

### 2.2. Benzoylecogonine (BEC)-BSA Conjugates and Indirect Competitive Enzyme-Linked Immunosorbent Assay (ELISA)

In 200 µL of dry, amine-free dimethylformamide (DMF), 2.76 mg of benzoylecgonine tetrahydrate (7.6 µM) were dissolved, and 18.6 µL (7.6 µM) of 0.43 M N-hydroxysuccinimide (NHS) dissolved in DMF were added. To the mixture, 11.7 mg N,N′-disuccinimidylcarbonate (DSC) (46 µM) were added [47] and allowed to incubate for 10 min. Subsequently, 1.2 µL (7.8 µM) of diisopropylcarbodiimide (DIC) were added, and the mixture was shaken for 20 h at 800 rpm at RT. The mixture was centrifuged, and the obtained supernatant was added to 1.27 mL (0.38 µM) of a 2 wt.% solution of BSA in 0.1 M sodium bicarbonate and incubated for 1 h at RT at 800 rpm. The solution was purified and desalted by an SEC column HiTrap™ Desalting, 5 mL (GE Healthcare) with a flow of 5 mL min^−1^. The eluate collection was controlled by the 280 nm signal and collected in 1 mL fractions, which were individually analyzed by MALDI-TOF-MS, and subsequently pooled and lyophilized (see Figure S1).

ELISA procedure: Each well of a 96-well plate was coated with 100 µL of 0.5 µg L^−1^ BEC-BSA (see Figure S2) and 0.5 µg L^−1^ BSA [46].

The plate was blocked with PBS with 0.1% of BSA (PBSB) for 75 min and washed. Subsequently, 75 µL of diluted cocaine in PBS ranging from 1 µM to 200 fM and 75 µL of 1:40,000 diluted antibody (IP3G2, approx. 0.24 µg L^−1^) in PBS were added as eight replicates and incubated for 75 min at RT in the dark.

After a washing step, 100 µL of 1:20,000 diluted (approx. 40 µg L^−1^) HRP-conjugated anti-mouse (H + L) IgG antibody in PBS with 0.5% BSA were incubated for one hour in the dark. The MTP was washed subsequently, and in each well, 50 µL of the chemiluminescent substrate was added and measured.

### 2.3. Manufacturing of the BEC-BSA Affinity Column and Cocaine Dilutions

Raw affinity columns were manufactured and prepared as described elsewhere [48]. Briefly, the column was cleaned, silanized with diethoxy(3-glycidyloxypropyl)-methyl silane and coated as described in Table S3. For the preparation of the BEC-BSA-affinity column, 4 mL of a 1 mg mL^−1^ BEC-BSA solution in 0.1 M Na_2_HPO_4_ pH 7.8 were incubated for one week at room temperature on the epoxy-functionalized raw column. The column was purged with 80% ethanol containing 20% water and stored under the same solution at 4 °C in the dark for several months without noticeable degradation in column performance. Cocaine hydrochloride was dissolved in methanol to a stock solution of 10 mM. For standards and spiking, it was further diluted in ethanol and PBSB as required.

### 2.4. Design and Synthesis of the IP3G2 Fluorophore Conjugate

Of the antibody stock solution (IP3G2), containing 3.75 mg mL^−1^ IP3G2 and 0.1 wt.% NaN_3_ in PBS, 53.3 µL (1.33 nM) were diluted with 46.7 µL of PBS-C (100 mM phosphate and 137 mM sodium chloride pH 7.8) to a final volume of 100 µL and a concentration of approx. 2 g L^−1^. A PD SpinTrap G-25 (Cytiva) was conditioned four times with 140 µL of PBS-C at 800 g and 4 °C. The diluted IP3G2 solution (100 µL) was transferred to the conditioned SpinTrap, and an additional stacking buffer of 40 µL PBS-C was added. The SpinTrap was centrifuged at 800 g for one minute at 4 °C, and the eluate, 140 µL, was collected.

1.68 µL (7.6 nM) of 4.5 mM Dy-654-NHS in DMF, prepared as described in [46], were added to the eluate, a six-fold molar excess, and shaken for 2 h at 800 rpm and 21 °C in the dark and subsequently stored for 96 h at 4 °C in the fridge. The conjugate was purified by size exclusion chromatography (SEC) with a PD SpinTrap G-25 (Cytiva) and conditioned with PBS as described above. To the 140 µL eluate, containing approx. 1.25 mg mL^−1^ IP3G2-Dy-654, 5 vol.% of 1:100 diluted ProClin300 was added as a preservative. The labeled antibody was stored in the dark at 4 °C until further use and remained stable for several months.

### 2.5. Fluorescence Detector, Fluidics, and Measurements

**Figure 2:**
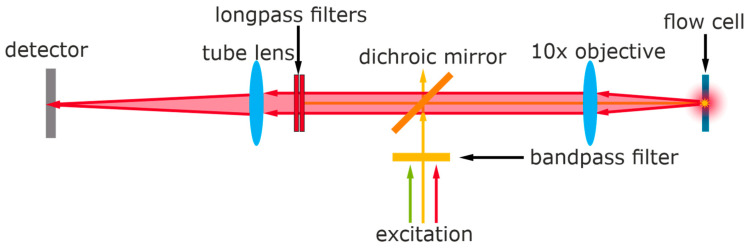
The light path of the detector setup with semiconductor laser excitation (yellow) and fluorescence (red) which is recorded as a signal by the Peltier-cooled CMOS detector.

The optical setup is based on an epifluorescence microscope setup (Figure 2). A semiconductor laser with a wavelength of approx. 638 nm is focused on the microfluidic flow cell by a microscope objective, and the same objective is used to collect the generated fluorescence. Two stacked long-pass filters and a dichroitic mirror remove light under 650 nm and allow only the fluorescence to reach the detector. Detailed plans of the setup, including a parts list, can be found elsewhere [46].

**Figure 3:**
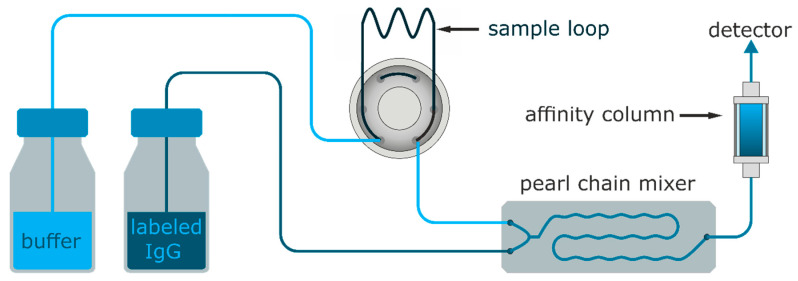
The fluidic setup for the online detection of cocaine. Injections were performed with a six-way-valve on the buffer channel for continuous flow injection. Both channels were mixed 1:1 with a dedicated mixer chip and flown through the BEC-BSA affinity column.

Cocaine detection and optimization of the injection volume: Cocaine was diluted to 500 pM in PBSB. A constant flow of 0.25 mL min^−1^ 1:40,000 diluted IP3G2-Dy-654 in PBSB was mixed 1:1 with a constant flow of 0.25 mL min^−1^ PBSB as a carrier buffer (see Figure 3). The injection valve was equipped with sample loops ranging between 50 and 500 µL. The cocaine sample (500 pM) was injected every six minutes.

Cocaine detection, dynamic range estimation:

(a) Cocaine was diluted from a stock solution to 100 to 3200 pM in 200 pM steps in PBSB. A constant flow of diluted IP3G2-Dy-654 in PBSB was mixed 1:1 with a constant flow of PBSB as described above. The injection valve was equipped with a sample loop of 200 µL, and the samples were injected every six minutes.
(b) Cocaine was diluted from a stock solution to 200 to 1000 pM in 200 pM steps in PBSB. A constant flow of diluted IP3G2-Dy-654 in PBSB was mixed 1:1 with a constant flow of PBSB as described above. The injection valve was equipped with a sample loop of 500 µL, and the samples were injected in cycles of six minutes.

Cocaine detection, determination of the limit of detection (LOD): Cocaine was diluted from a stock solution from 100 to 200 pM in 100 pM steps in PBSB. A constant flow of diluted IP3G2-Dy-654 in PBSB was mixed 1:1 with a constant flow of PBSB as described above. The injection valve was equipped with a sample loop of 500 µL, and the samples were injected in cycles of six minutes as triplicates.

**Figure 4:**
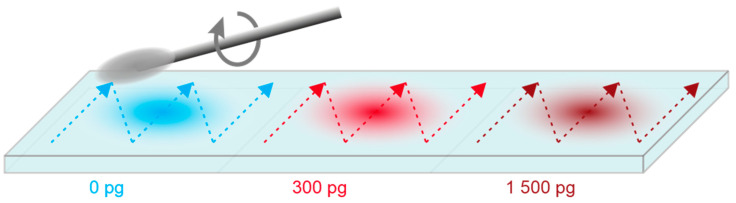
Polymer slide with individual sample squares containing 0, 300, and 1500 pg cocaine. Performed swiping pattern with the cotton swab is shown (colored arrows).

Cocaine detection with surface sampling:

Sample Preparation: Cocaine was diluted from a stock solution to a 1 µM solution in ethanol. Three individual polymer slides (TOPAS, 10000443, Microfluidic ChipShop) of 75.5 × 25.5 × 2 mm labeled as S1 to S3 were cleaned with pure water and each divided into three equal squares of approx. 25 × 25 mm (see Figure 4). Each sample area on every substrate was spotted once. The upper square was spotted with 5 µL of cocaine-free absolute ethanol, referred to as blank in the following, resulting in a wetted circle of approx. 20 mm and allowed to dry. The central square was spotted with 1 µL of 1 µM cocaine (300 pg cocaine) in ethanol, resulting in a wetted circle of approx. 15 mm and allowed to dry. The lower square was spotted with 5 µL of 1 µM cocaine (1500 pg cocaine) in ethanol, resulting in a wetted circle of approx. 20 mm and allowed to dry. The dried substrate slides S1, S2, and S3 were stored in 50 mL vials until the surface wipe test was performed.

Swab preparation: Nine consumer-grade cotton swabs (Cien, EAN 2047 6830) were cut to size by removing the second cotton head. Subsequently, the swabs were washed for 20 min with 30 mL PBSB, for five minutes in 30 mL pure water, and finally rinsed with PBSB. The conditioned swabs heads were squeezed against the vial surface to remove excess buffer while remaining wet to the touch and placed in a 50 mL vial until the surface wipe test was performed.

Internal Calibration: Cocaine was diluted from a stock solution from 250 to 1000 pM in 250 pM steps in PBSB. A constant flow of diluted IP3G2-Dy-654 in PBSB was mixed 1:1 with a constant flow of PBSB as described above. The injection valve was equipped with a sample loop of 500 µL, and the samples were injected every six minutes. After the calibration was injected, the surfaces wipe test was performed.

Sampling and measurement: A conditioned swab was guided at an angle of approx. 15 degrees over the sample square of approx. 25 × 25 mm on the slides S1, S2, or S3. The swiping pattern consisted of four repetitions of three up and down motions while slightly rotating the swab (see Figure 4) and required about 20 s for each square. The cotton swab head was removed and placed in a 5 mL tube containing 3 mL of running buffer (PBSB) and vortex for 15 s at 2700 rpm. Of the solution approx. 2.5 mL were aspirated with a plastic syringe, a 17 mm 0.2 µm cellulose filter was added, and approx. 2 mL were injected to purge and fill the 500 µL sample loop. The whole procedure from swiping (~20 s), over removing the cotton head, and extracting (~30 s) to complete the injection required approx. 90 s in total and was performed for every sample on every substrate.

### 2.6. Data Evaluation

Biosensor: The raw data was recorded as a sequence of .fits-image files with the SharpCap software (https://www.sharpcap.co.uk, accessed on 1 September 2021,version 3.0.4074.0) with a fixed exposure time of 5000 ms, a gain of 0, and a sensor temperature of −5 °C. The position of the laser center (see Figure S4) on the flow cell remained very stable over time; only a minor shift within a few pixels was observed. Therefore, a 50 × 50-pixel area around the laser center was defined as the region of interest (ROI). In order to evaluate each frame, all pixels within the ROI were sorted according to their intensity. The highest three pixels of the ROI were discarded to account for hot pixels or cosmic rays, and the mean of the following five pixels was determined, used as the intensity of the frame, and exported as a .txt file. This was performed semi-automated for all frames by a python script described in the SI (see Figures S15–S17) and included in the Supplementary Materials.

In order to determine the signal intensities of the injected samples, the evaluated data was smoothed with a Savitzky-Golay filter (*n* = 11 and *p* = 2), and the 1st derivative was calculated. To determine the peak maximum and end of signal growth, for each injection peak, the first frame (f_n_) to show a negative 1st derivative was picked. To determine the peak height, f_n_ and the frames prior and subsequent of f_n_ were used to calculate the mean and the standard deviation for the injected sample. Besides the signal height, the 1st derivative of the signal was determined based on the moving average smoothed (*n* = 12, one minute). The maximum of the 1st derivative for each sample was picked.

Indirect competitive ELISA: The intensities were fitted by a four-parameter logistic function to determine the test midpoint (IC50). To determine the relative error of concentration, a precision profile according to Hoffmann et al. [49] was calculated, and the limit of detection (LOD) was determined based on a relative error of concentration of 30% [50].

## 3. Results

### 3.1. BEC-BSA Synthesis and Conjugate Characterization

**Figure 5:**
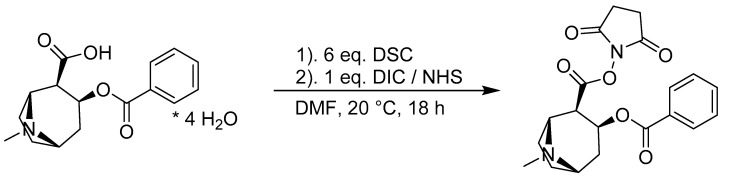
Chemical drying of the benzoylecgonine tetrahydrate (* 4 H_2_O) solution with N,N′-disuccinimidylcarbonate (DSC) followed by activation with diisopropylcarbodiimide (DIC) and N-hydroxysuccinimide (NHS) to obtain the BEC-NHS ester.

In order to synthesize the hapten for the affinity column and the indirect ELISA, the NHS activation route with BEC was chosen [42]. Benzoylecgonine tetrahydrate was dried with DSC in DMF to remove water [47] and subsequently activated with DIC and NHS (see Figure 5). DIC was chosen over DCC for the convenience of handling, as it is a liquid at RT and showed similar performance to N,N′-dicyclohexylcarbodiimide (DCC) in preliminary experiments. Size-exclusion chromatography (SEC) was performed to separate the protein-containing fraction from unbound BEC and hydrolyzed NHS. The fractions identified by UV absorbance at 280 nm were collected and analyzed individually by MALDI-TOF-MS (Figure S1). The initial 20-fold molar excess of BEC per BSA molecule leads to a degree of labeling (DOL) of the conjugate of approximately seven BEC per BSA. The fractions containing the majority of the product and showing a DOL of approx. 7 and (Figure S2) were pooled and lyophilized.

### 3.2. Antibody-Labelling and Indirect Competitive ELISA

**Figure 6:**
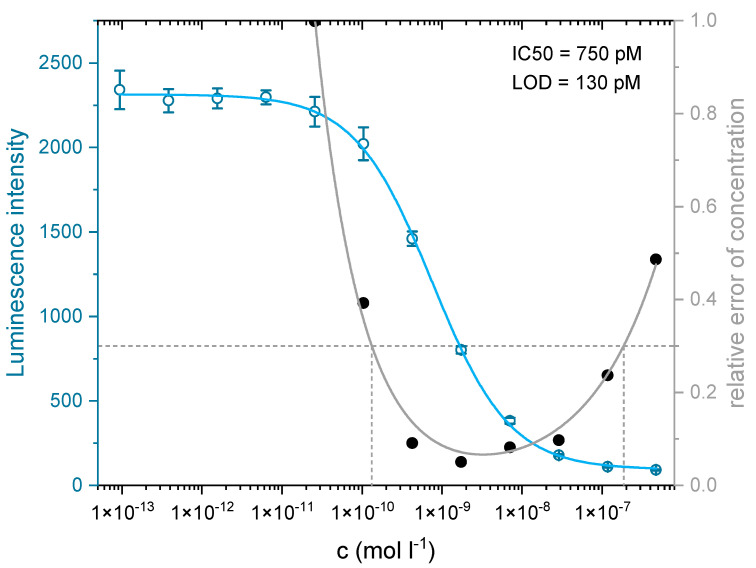
An indirect competitive ELISA and precision profile (black dots, grey line) for the clone IP3G2. The IC50 was determined to be 750 pM, and a working range of 130 pM to 190 nM was obtained.

As the biosensor relies on fluorescence detection, the chosen label of the antibody is of considerable importance. The desired label should combine a strong absorbance, a high quantum yield, and photostability, along with excellent water solubility and low unspecific binding. Also, the label must not show any cross-reactivity with the antibody IP3G2, which was confirmed in preliminary experiments (see Figure S5). Due to common autofluorescence in the blue spectral region, the red label Dy-654 was chosen for this application. The dye is based on a cyanine backbone with four sulfonic acid groups (see Figure S6, which results in highly hydrophilic behavior. The dye works well with the used excitation source of 638 nm and has proven to display negligible non-specific binding to epoxy-functionalized glass substrates [51] or trinitrophenyl-BSA affinity columns [46]. The degree of labeling of the IP3G2-Dy-654 conjugate was determined with MALDI-TOF MS to be approx. 5 (see Figure S7), and the concentration of the conjugate was estimated to be approx. 1.25 g L^−1^. The affinity of the clone IP3G2 to cocaine was investigated with an optimized indirect competitive ELISA with chemiluminescence detection. The test midpoint was determined to be approx. 750 pM or 230 ng L^−1^, respectively (see Figure 6), which is in agreement with the datasheet and literature stated values for the clone IP3G2 of 260 pM [42] with direct competitive ELISA. Based on the precision profile [49], a limit of detection (LOD) [50] of 130 pM or 40 ng L^−1^ was determined. As different suppliers also sell the clone IP3G2 under various names (e.g., MAB4029, G45132M, and IP3G2), a unique antibody fingerprint [52] was generated to ensure the identity of the clone (see Figure S8) and allow for traceability in future projects.

### 3.3. BEC-BSA Column Performance: Influence of the Injection Volume and the Reaction Time

**Figure 7:**
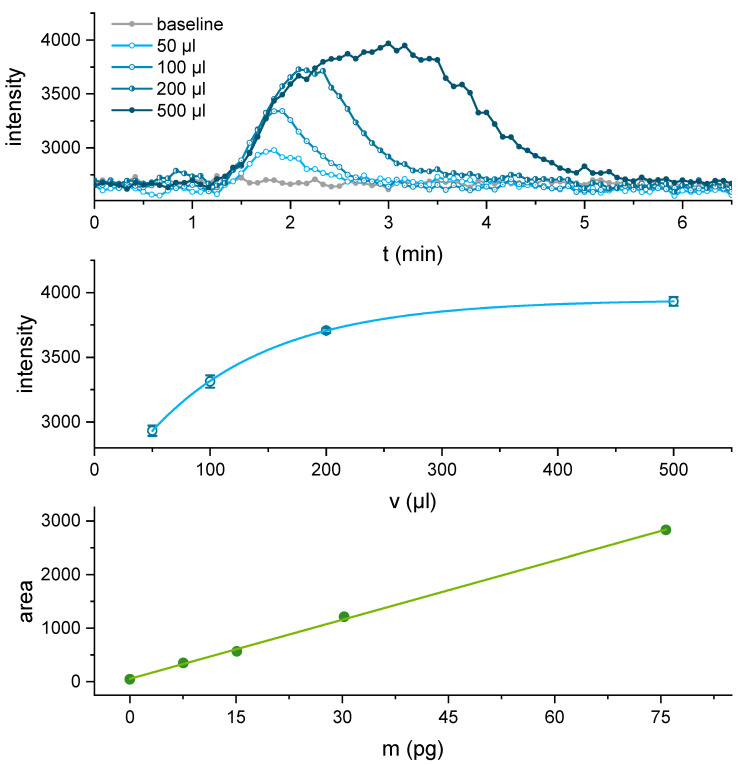
Signals of injection of 50 to 500 µL of 500 pM cocaine (**top**) and obtained signal intensities with standard deviation with an asymptotic fit (**middle**). The peak area is plotted against the injected amount of cocaine (pg) with a linear fit (**bottom**).

The BEC-BSA column showed high antibody retention of approx. 80% at the working conditions of approx. 12.5 µg L^−1^ of IP3G2-Dy-654 and a flow rate of 0.5 mL min^−1^. In preliminary experiments, three commercially available microfluidic micromixers, a “herringbone-mixer”, a “vortex-mixer”, and a “pearl-chain-mixer” were evaluated for their performance at the given flowrates used as shown in Figure 3. In this setup, the micromixer “pearl chain mixer” Chip (10000759, microfluidic ChipShop, Jena, Germany, see Figure S9, proved to be the most suitable choice for this application with a wide range of mixing ratios and homogenous mixing at a 1:1 ratio (see Figure S10). In order to identify the optimal sample volume for this setup, sample loops between 50 and 500 µL were tested with a sample solution of 500 pM (150 ppt) of cocaine, injecting amounts of 8, 15, 30 to 76 pg of cocaine into the system (see Figure 7).

For every injection volume from 50 to 500 µL, a signal above the background was obtained. While peak growth remained very similar, with increasing injection volume, the position of the peak maxima shifted to longer times. Also, the peak shape became less sharp and almost reached a plateau for the 500 µL sample loop. If we consider the internal volume of the affinity column of approximately 400 µL, this signal saturation is reasonable. Therefore, to achieve the highest sensitivity, a sample loop of at least 500 µL should be used. The reaction time of the system is determined by the first measurement to exceed the background noise, which was determined as 3 s of the blank signal. Independently of the chosen sample loop volume, the first signal to exceed this threshold is typically detected after 90 s. Considering the flow rate of 0.5 mL min^−1^, this represents a volume of ~0.75 mL, which about equals the expected dead volume of the system of 0.7 mL.

### 3.4. Dynamic Range and Limit of Detection for Cocaine Detection

To estimate the usable detection range for the cocaine online method, samples from 200 pM (60 ppt) to 1000 pM (300 ppt) cocaine were injected, delivering a total amount of 30 to 150 pg of cocaine with the 500 µL sample loop (see Figure S11). The signal suggests a linear range below 500 pM and an asymptotic behavior at higher concentrations, which is in accordance with TNT measurements previously described for this assay type [31,46].

The limit of detection for this biosensor was determined by injection of triplicates of 0 pM, 100 pM (30 ppt), and 200 pM (60 ppt) cocaine, respectively, which is equivalent to an absolute injected amount of 0, 15, and 30 pg cocaine.

**Figure 8:**
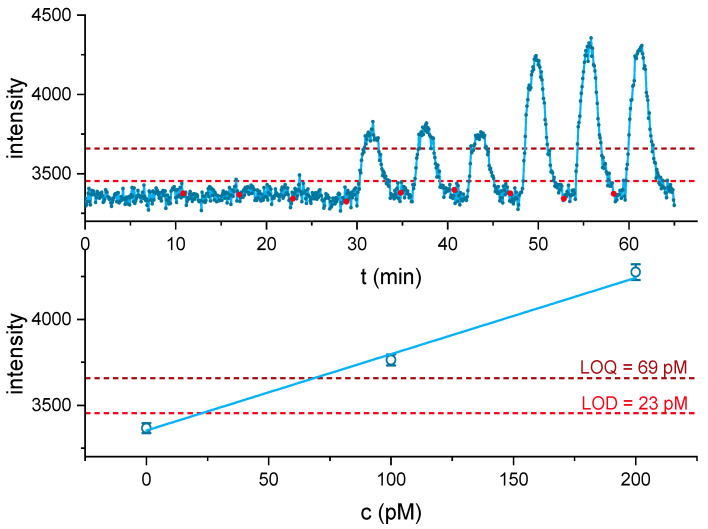
Injection of 0, 100, and 200 pM cocaine (**top**) with highlighted injection start (red dots). The obtained intensities were evaluated (**bottom**) and linearly fitted. The limit of detection and quantification were determined as 3 s of the baseline with three blank injections and were highlighted in both graphics. The limit of detection (LOD) was determined to be 23 pM (7 ppt), a total amount of 4 pg cocaine. The reaction time, before a sample of 100 pM cocaine was sufficient to exceed background, was determined to be approx. 1.6 min and peak maximum was reached after approx. 3 min.

Evaluation of the signal heights resulted in a LOD of 23 pM or 7 ppt (see Figure 8); if the 1st derivative of the 12-point moving average smoothed data is evaluated, a LOD of 28 pM is obtained (see Figure S12).

### 3.5. Surface Sampling, Reaction Time, and Analyte Recovery

An internal calibration with standards from 250 to 1000 pM cocaine, including three blanks, was performed directly before the surface wipe measurements. The calibration was used to determine the concentration of the nine wipe samples, collected from three separate polymer sides (S1, S2, and S3), each containing 0, 300, or 1500 pg of cocaine. The calibration resembled an asymptotic behavior as previously observed (Figure S11), and the LOD was determined to be 24 pM, which is in good agreement with the detailed determination of the LOD based on the triplicates (see Figure 8).

**Figure 9:**
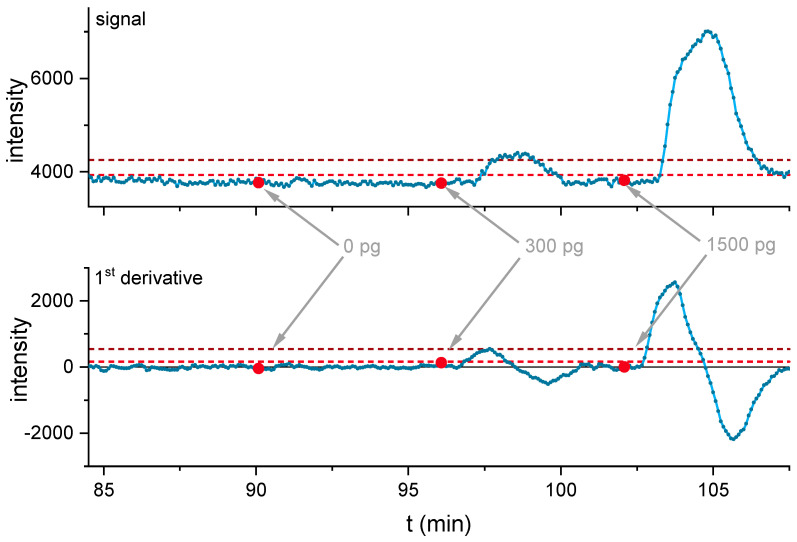
Signals of the first wiped slide, with samples spiked from 0, 300 to 1500 pg cocaine equivalents (**top**) and 1st derivative of the 12-point moving average smoothed signal (**bottom**). The limit of detection (light red dashed line) and the limit of quantification (dark red dashed line) were highlighted and are based on the baseline of the blank samples.

**Figure 10:**
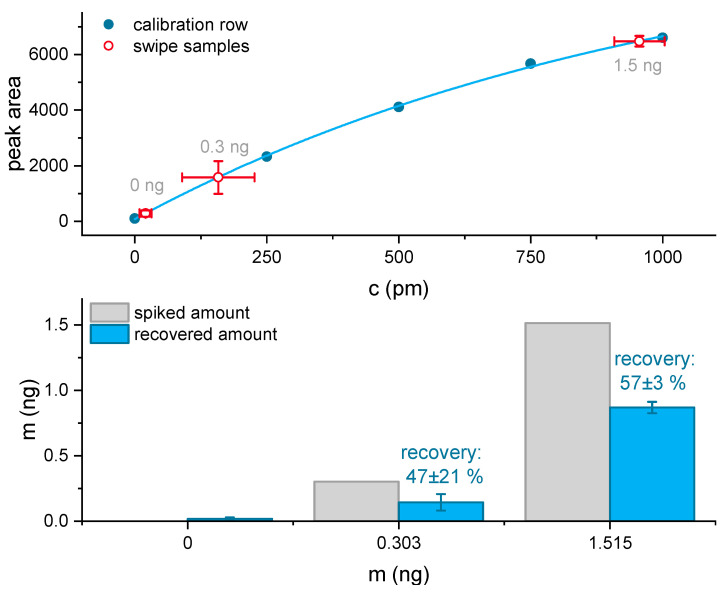
Calibration from 250 to 1000 pM cocaine based on the peak area with an asymptotic fit. The mean and the standard deviation of the 0, 0.3, and 1.5 ng triplicate samples were highlighted (**top**). Comparison of the spiked amount of cocaine to the amount of cocaine (recovery) determined by the wipe test based on the peak areas (**bottom**).

Due to the high sample concentration in the 1500 pg wipe sample, the next subsequent 0 pg wipe sample suffered from a slightly increased baseline due to peak tailing (see Figure S13). In order to increase detection robustness, the signal was smoothed by a 12-point (one minute of measurement time) moving average, and the 1st-derivative was determined (see Figure 9 and Figure S13). For the 0 pg blank sample of the first sampled surface, neither the LOD of the signal height (LOD = 24 pM) nor the LOD of the 1st derivative (LOD = 31 pM) was exceeded. In contrast, all cocaine spiked surfaces were correctly identified (see Figure 9), exceeding LOD and LOQ. Based on the peak areas of the calibration samples from 250 to 1000 pM and the corresponding asymptotic fit, the cocaine concentration in the wipe samples was determined. Also, the overall recovery efficiency of the sampling procedure was estimated (see Figure 10).

In the surface wipe test, all positive samples of 300 pg and 1500 pg cocaine exceeded the LOQ of the signal intensity and the 1st derivative based on the smoothed data. Signals started to exceed the background as early as 90 s after injection, showing the maxima in the 1st derivative after 95 s (Figure S14). Based on the peak areas, the 0 pg sample 18 ± 10 pg, for the 300 pg sample 140 ± 62 pg (47 ± 21%), and for the 1500 pg sample, 870 ± 44 pg (57 ± 3%) recovery were determined (Figure 10).

## 4. Discussion

Based on kinetic competition, a highly sensitive biosensor for the detection of benzoyl-methyl ecgonine (cocaine) with a sensitivity of <10 ppt (ng L^−1^) in 90 s was demonstrated. Compared to traditional ELISA, in this biosensor, the analyte is incubated in a homogeneous solution with the labeled high-affinity antibody, which is a fast process. This incubation step is continuously performed in a microfluidic mixer chip, eliminating further liquid handling, reducing analysis times, and allowing for online measurements. The high capacity and stability of the monolithic affinity column combined with the minute consumption of fresh antibodies result in good long-term measurement capability combined with reliable performance. The sensor exhibits a calibration curve with a positive slope with high sensitivity at low analyte concentrations. Compared to other recent immunochemical methods [21], the biosensor has a shorter response time of approx. 1.5 min. After 3 min, the maximal signal height is reached. As these delays are a result of the internal dead volume of the system, either higher flow rates or shrinking of the setup, especially the affinity column, would further reduce assay time if required.

The affordable cost of the in-house developed affinity columns and the laser-induced fluorescence detector (LIF) enables a broad range of applications, as expensive high-end components are not required. Additionally, the minute antibody consumption, below 0.5 µg per hour, the low energy consumption, and the non-use of organic solvents and expensive reagents result in extraordinary low running costs of < 1 EUR/hour.

For the liquid handling, high-performance syringe pumps are used to deliver quasi pulsation-free flow to allow optimal carrier-buffer and antibody-buffer mixing in the microfluidic chip. Samples are introduced into the flow by a conventional 6-port injection valve with a sample loop. Continuous sample introduction for true online monitoring, while possible, was not performed yet. As an additional application example, a surface wipe test was demonstrated to detect cocaine residues (0.3 and 1.5 ng) on plastic surfaces. The sampling procedure required about 90 s, and buffer-soaked cotton swabs were used. Positive signals exceeding the background were identified for all cocaine-containing samples as early as 90 s after sample injection. For the data, either the signal intensities or the 1st-derivative could be used to evaluate the signals with a similar limit of detection. For the wiping procedure, recoveries of 40 to 50% were obtained. With improved extraction methods and or lower extraction volumes, cocaine amounts down to 0.1 ng should be detectable. For swab tests analyzed with an LC-MS/MS, limits of quantitation of 1 ng/swab were reported in a recently published paper [53]. This means that our biosensor system shows a comparable or even superior performance in relation to a lab-based LC-MS/MS approach.

## 5. Conclusions

Based on a benzoylecgonine-conjugated monolithic affinity column and the high-affinity antibody IP3G2, a biosensor for the trace detection of cocaine was developed. Due to the utilization of a microfluidic mixer and flow cell chips, continuous measurement was achieved, while system costs and dead volume were held minimal. Compared to the optimized indirect ELISA (assay time > 150 min, LOD 40 pM cocaine), the biosensor was able to achieve higher sensitivity (LOD 23 pM) within a much shorter time frame, reaching full signal height three minutes after sample introduction and indicating the presence of cocaine after just 90 s. Also, compared to ELISA, measurements are performed quasi continuously, and the experimental effort is greatly reduced. Additionally, wipe sampling of cocaine-spiked plastic surfaces was demonstrated, identifying cocaine residues of 0.3 and 1.5 ng within three minutes. The affinity columns and labeled antibodies remained stable for months, and overall reagent consumption is very low. The incorporation of a micro-mixer allowed for measurements with an uninterrupted flow. This reduced baseline artifacts, leading to reduced limits of detection. The injection volume optimization showed that sample volumes down to 50 µL are sufficient. Hence, a faster sampling frequency is possible if required. It can be concluded that such biosensor systems show very high potential for drug and explosive screening, for example, at airports, harbors, train stations, and other high-risk areas. Furthermore, continuous screening for high-volume drug smuggling in shipping containers might be feasible. An additional advantage is the high flexibility of the approach, which can be applied for all analytical targets, including small molecules, for which high-affinity antibodies are available or can be developed.

## Data Availability

Not applicable.

## Supplementary Materials

The following are available online at https://www.mdpi.com/article/10.3390/bios11090313/s1, Figure S1. SEC purification of the BEC-BSA conjugate Figure S2. MALDI-TOF MS of BEC-BSA conjugate. Figure S3. Fluorescence image with active laser spot in the center of the microfluidic chip used as a flow cell. Figure S4. Competitive indirect ELISA of IP3G2 with cocaine and the label Dy-654. Figure S5. Structure of the label Dy-654 with a cyanine backbone and four sulfonic acid groups. Figure S6. MALDI-TOF MS of the antibody IP3G2 and the labeled antibody IP3G2-Dy-654. Figure S7. MALDI-MS fingerprint of IP3G2 antibody (tryptic digest). Figure S8. Micro mixer “pearl chain mixer”. Figure S9. Concentration steps of Dy-654. Figure S10. Online injection of cocaine and evaluated data with the asymptotic fit. Figure S11. 1st Derivative of the 12-point moving average smoothed intensities for triplicate injections of cocaine. Figure S12. Comparison of the signal intensities and the 1st derivative of the 12-point moving average smoothed intensities for all three sample slides. Figure S13. 1st Derivative of the 12-point moving average smoothed intensities of the internal calibration. Figure S14. Source directory with the evaluator.py script. Figure S15. Vertically flipped raw image with highlighted laser center position. Figure S16. The path to the raw data with all required input files. Table S1. Preparation of the Benzoylecgonine-(BEC)-BSA column.
