# Supplementary material for: Cocaine Detection by a Laser-Induced Immunofluorometric Biosensor
Source: Biosensors (Basel). 2021 Sep 3;11(9):313. doi: 10.3390/bios11090313 (PMC8466613; doi:10.3390/bios11090313)
Supplement: Supplementary file 1 [file biosensors-11-00313-s001.zip › Biosensors-1329158 SM/Supplementary Materials.pdf]

biosensors

MDPI

Supplement

Cocaine Detection by a Laser-induced Immunofluorometric Biosensor

Martin Paul, Robert Tannenberg, Georg Tscheuschner, Marco Ponader and Michael G. Weller *
* Correspondence: michael.weller@bam.de, Tel.: +49-30-8104-1150

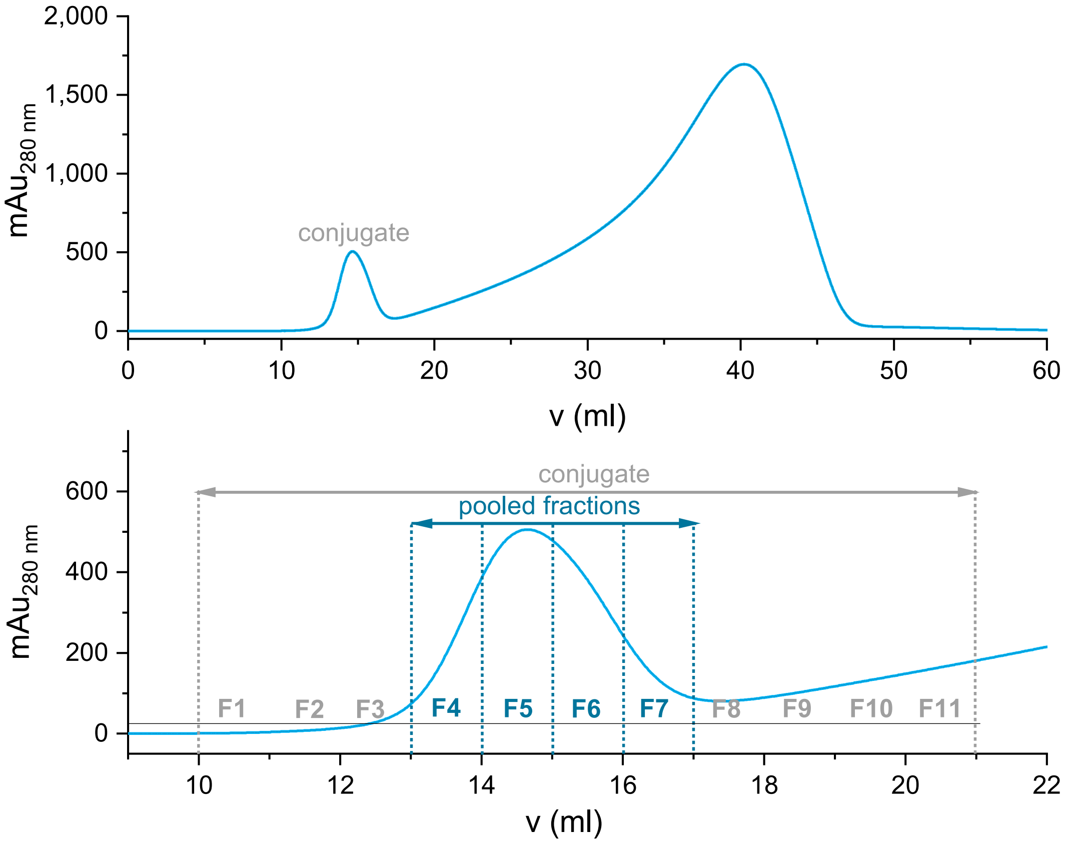

Figure S1. SEC purification of the BEC-BSA conjugate (top) with highlighted fractionation of the conjugate (bottom). Fractions were individually evaluated with MALDI-TOF-MS, and conjugate-containing fractions were pooled (F4-F7).

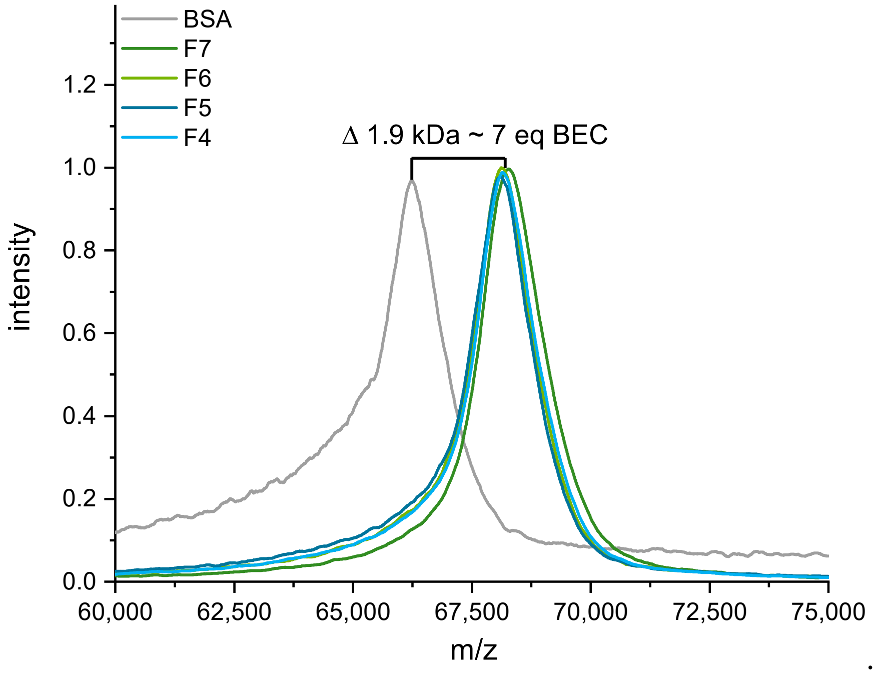

Figure S2. MALDI-TOF-MS of the pooled fractions (F4-F7) of the BEC-BSA conjugate compared to unmodified BSA (grey). A degree of labeling (DOL

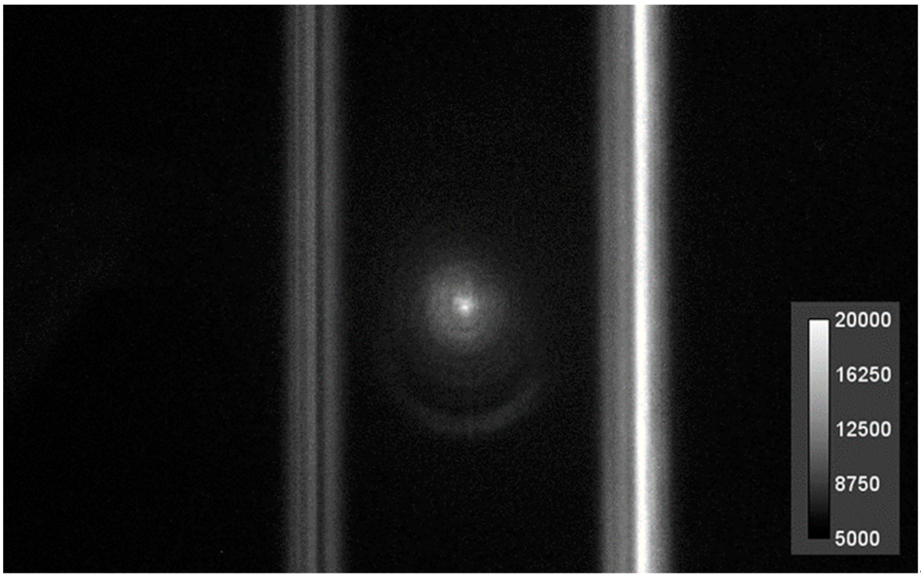

Figure S3. Fluorescence image as obtained by the detector with active laser spot in the center of the microfluidic chip used as a flow cell.

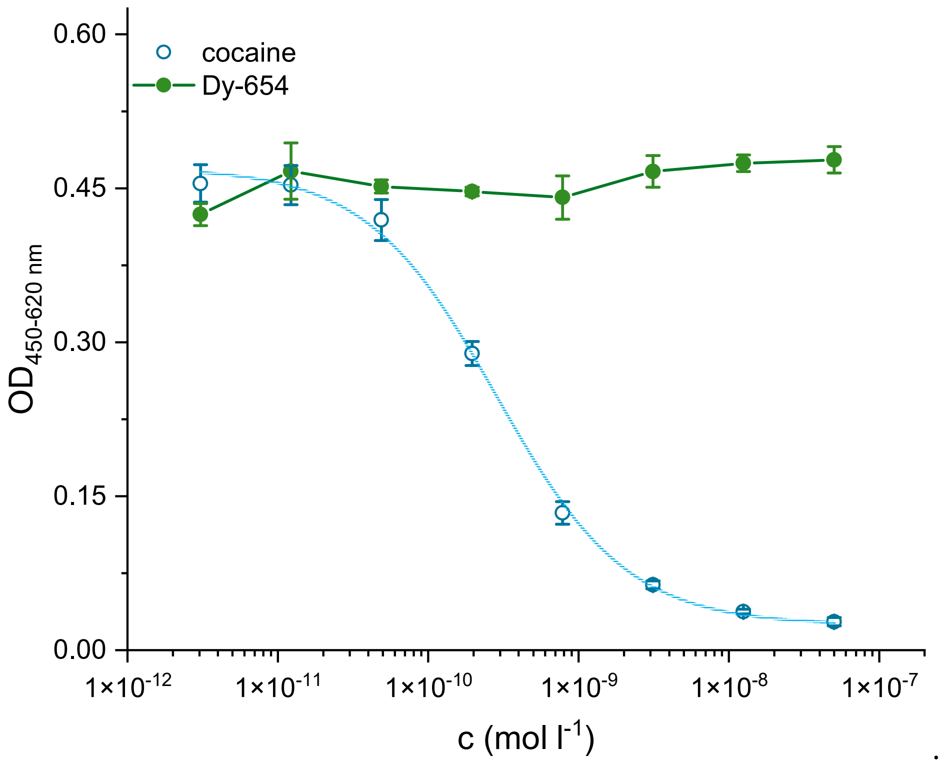

Figure S4. Competitive indirect ELISA of IP3G2 with cocaine and the label Dy-654. For the label Dy-654, no inhibition was observed.

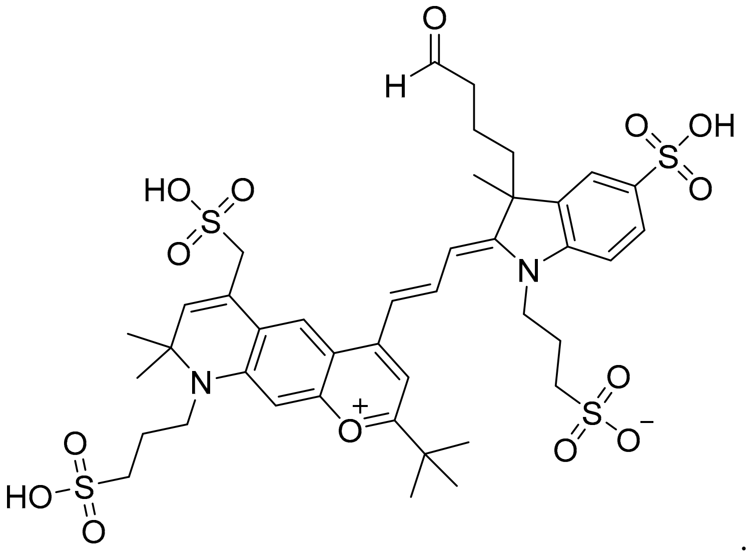

Figure S5. Structure of the label Dy-654 with a cyanine backbone and four sulfonic acid groups (Formula kindly provided by Dyomics, Jena, Germany).

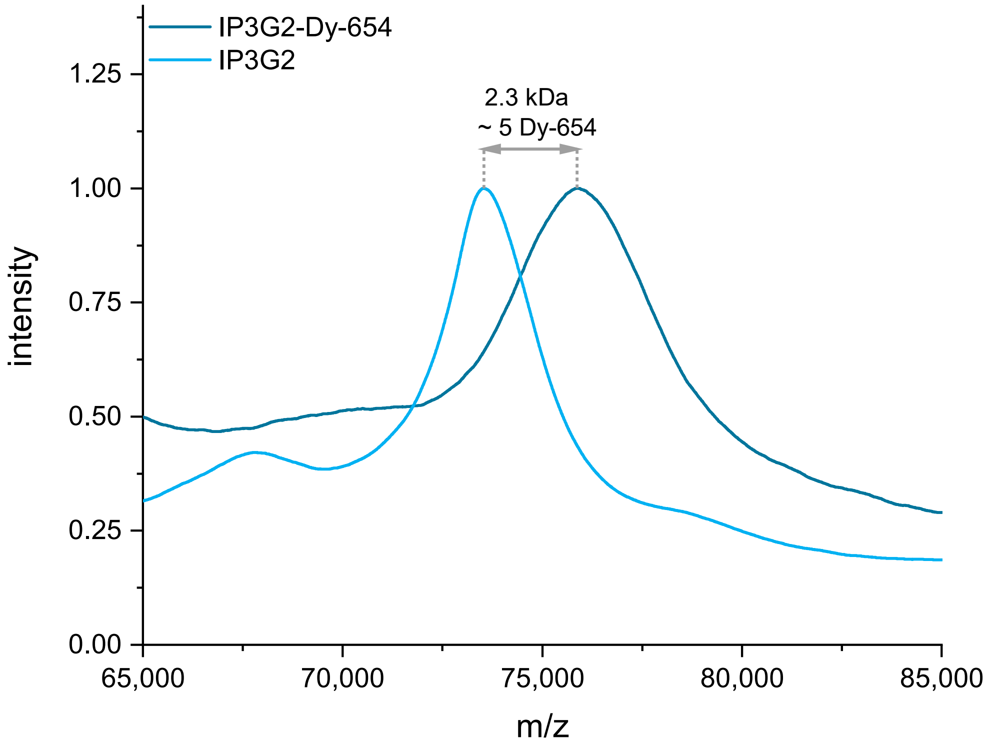

Figure S6. MALDI-TOF MS of the antibody IP3G2 (light blue) and the labeled antibody IP3G2-Dy-654 (dark blue) with a degree of labeling (DOL) of approx. 5.

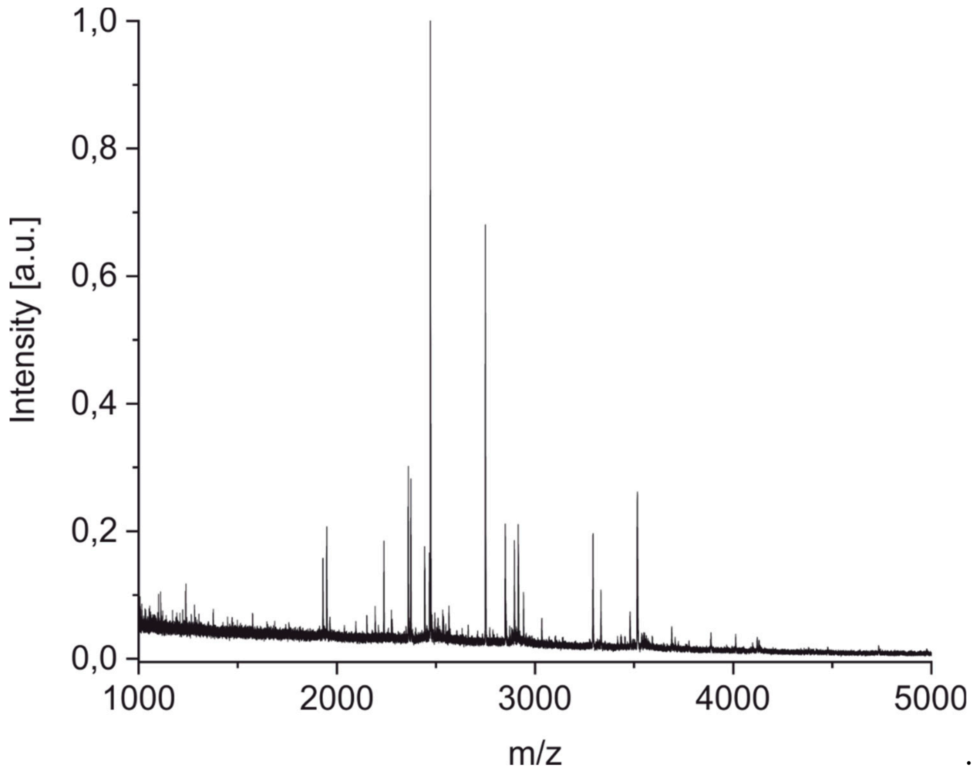

Figure S7. MALDI-TOF MS fingerprint of IP3G2 antibody against benzoylecgonine/cocaine (tryptic digest).

10 µg of antibody were incubated for 15 minutes with 0.1 mM tris(2-carboxyethyl)phosphine and 0.1 M tris(hydroxymethyl)aminomethane buffer (pH 7.8) at 99 °C in a total volume of 50 µL. After cooling to room temperature, trypsin was added in a 1:100 (w/w) ratio relative to the antibody and incubated for another 15 minutes at 55 °C. The sample was cleaned up by performing microscale solid-phase extraction in a pipette tip. MALDI measurement was performed on a Bruker Autoflex MaX in reflector mode using 2,5-dihydroxyacetophenone as MALDI matrix. These peptide masses may be used for identification purposes. It has to be noted that this antibody is based on ascites technology and hence may contain non-related IgG.

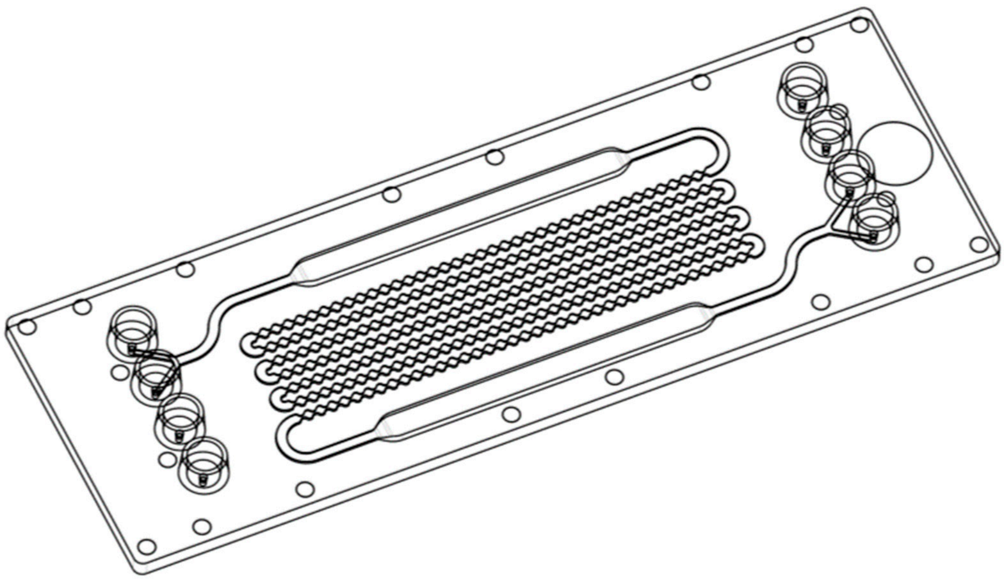

Figure S8. Micro mixer "pearl chain mixer" 10000759 (Technical drawing kindly provided by microfluidic ChipShop, Jena, Germany).

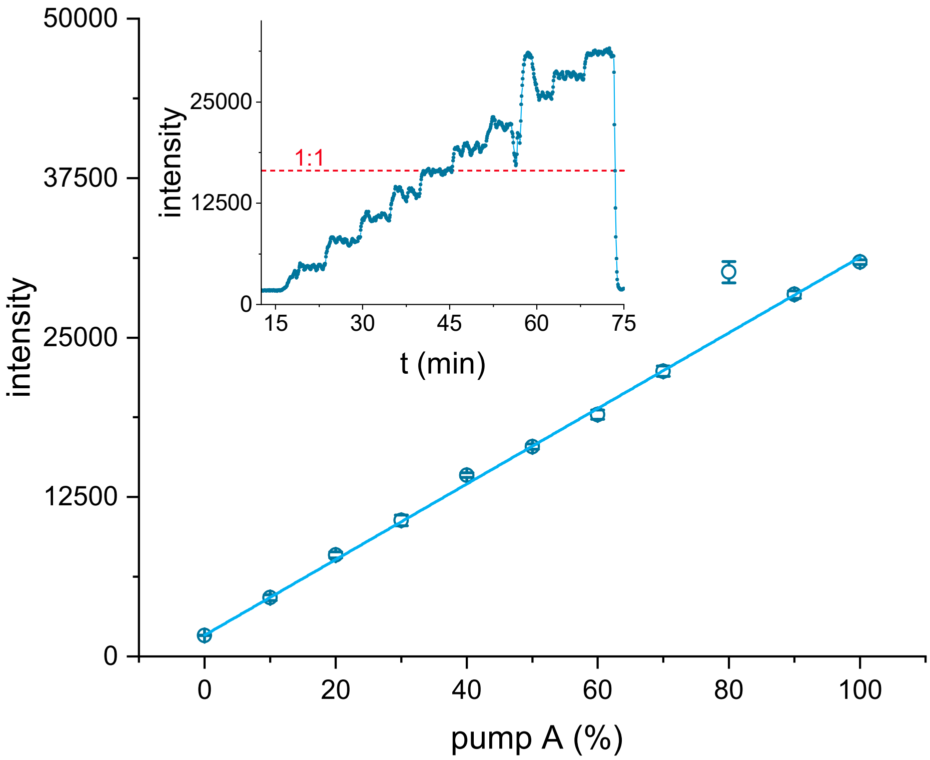

Figure S9. Concentration steps of 500 pM Dy-654 (pump A) and buffer PBS (pump B). The total flow rate is held constant at 0.5 mL min^{-1}, and the relative amount of A is increased in 10 % steps until 100 % is reached. The 1:1 mixture is highlighted red (insert). The obtained intensities are linearly fitted; the outlier at 80 % originates from a syringe exchange.

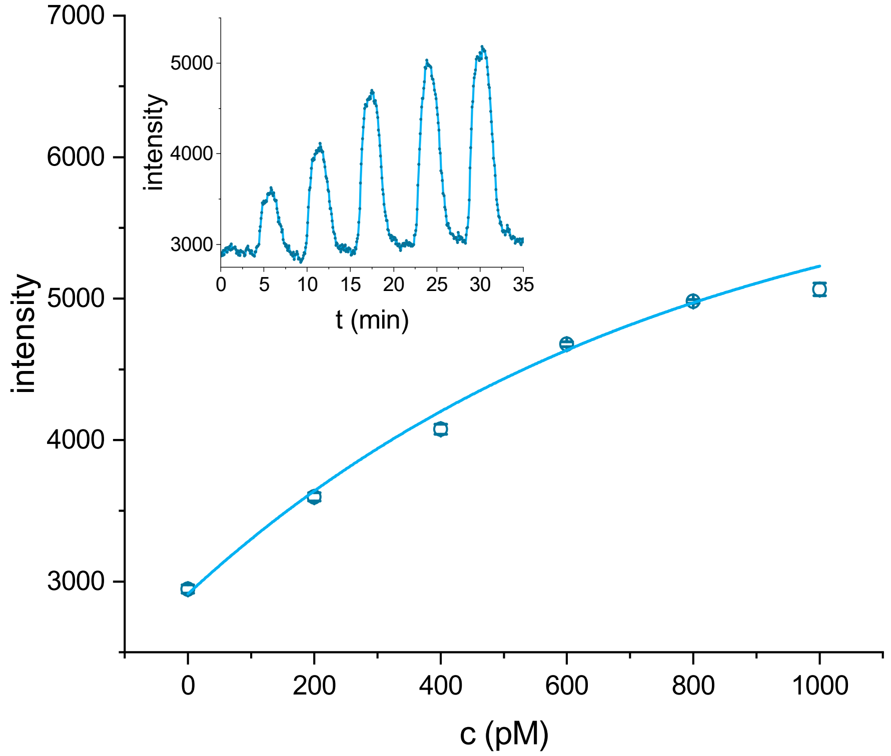

Figure S10. Online injection of 200 to 1000 pM cocaine (insert) and evaluated data with the asymptotic fit.

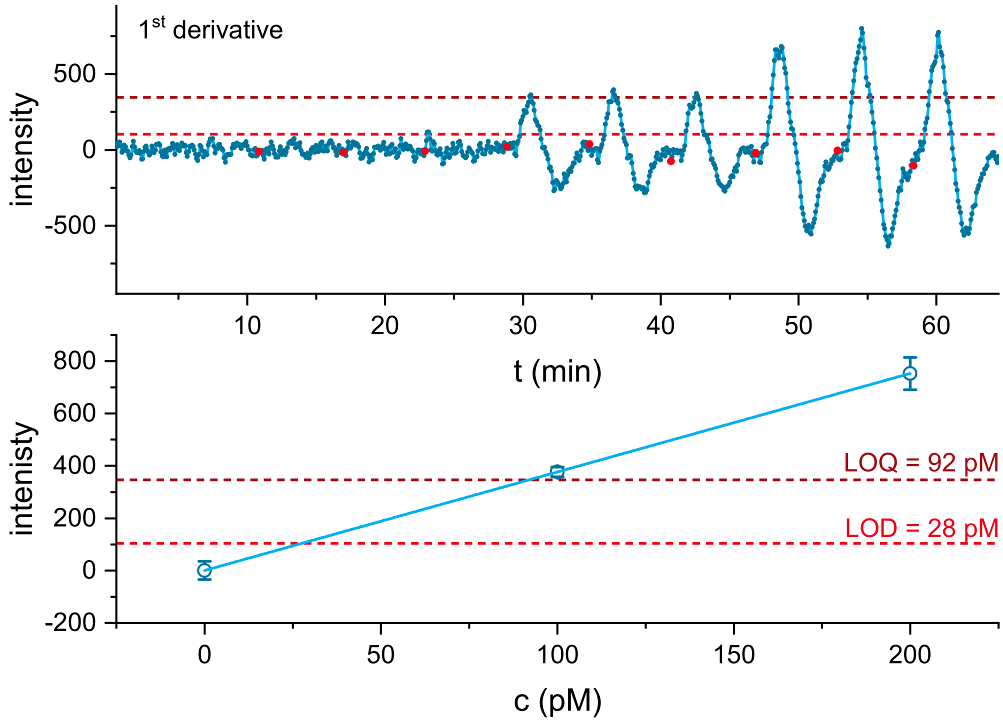

Figure S11. 1st Derivative of the 12-point moving average smoothed intensities for triplicate injections of 0, 100, and 200 pM cocaine (top) and linear fit with LOD and LOQ based on the baseline (bottom).

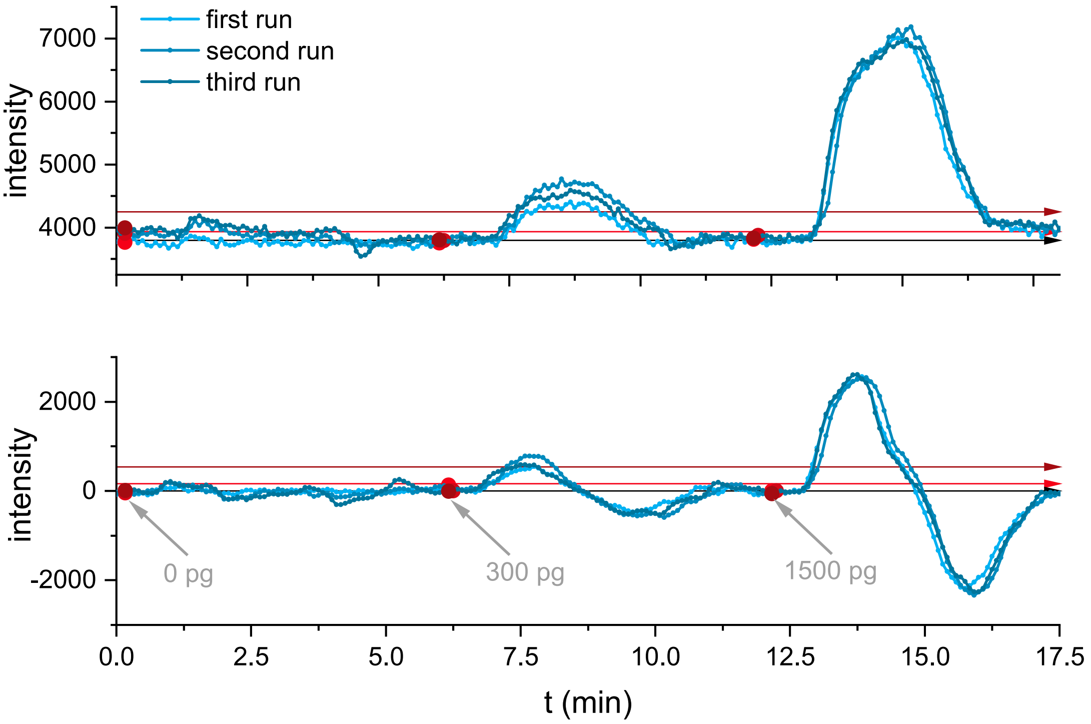

Figure S12. Comparison of the signal intensities (top) and the 1st derivative of the 12-point moving average smoothed intensities (bottom) for all three sample slides. The mean intensity of the background (black line), the limit of detection (light red line), and the limit of quantification (dark red line) are highlighted along with the injection starts (red dots).

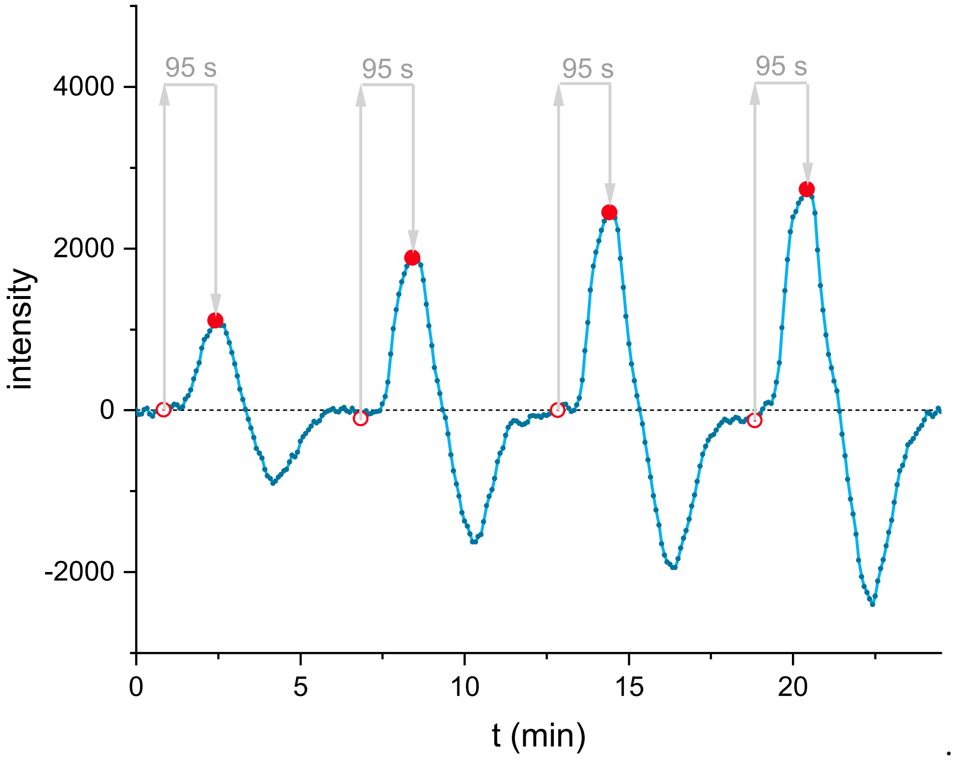

Figure 13. 1st Derivative of the 12-point moving average smoothed intensities of the internal calibration from 250 to 1000 pM cocaine. The injection (hollow red dot) and the maxima (red dot) are very reliably 19 frames or 95 seconds apart.

2. Data supplement

2.1 Sensor Data Evaluation

With Python 3.7 in the Spyder 3.2.2 by the Anaconda environment, the data evaluation of the fluorescence signals was performed. The script supplied in the data appendix with some sample files is intended for exactly this application. In the repository of the captured data (sequence of .fits, raw images), the following .txt files are copied: a "hotpixel_1920_1200.txt" file, which includes all known hot pixels of the sensor, and the roi_region_x_y.txt file, which includes the region of interest (ROI) coordinates of the laser spot.

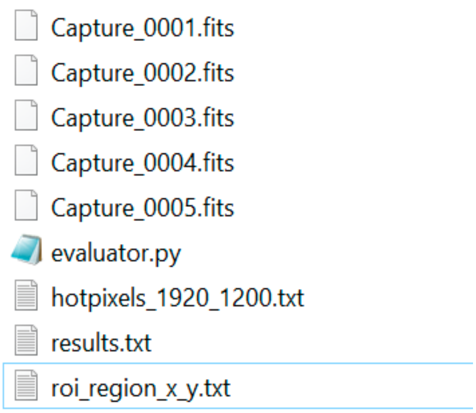

Copyright: © 2021 by the authors. Licensee MDPI, Basel, Switzerland. This article is an open access article distributed under the terms and conditions of the Creative Commons Attribution (CC BY) license (http://creativecommons.org/licenses/by/4.0/).

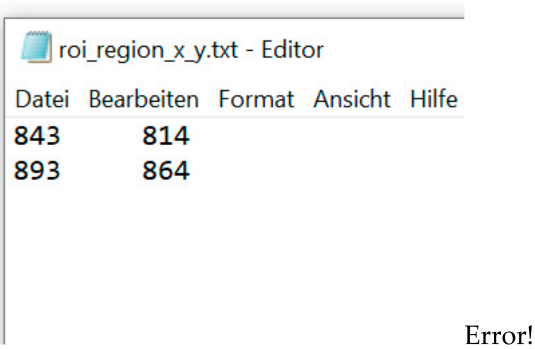

Error! Bookmark not defined.

Figure S14. Source directory with the evaluator.py script, the sequence of raw files (Capture_001-005.fits), the hot pixel file (hotpixels_1920_1200.txt), and the laser region of interest file roi_region_x_y.txt (left). Opened roi_region_x_y.txt file (right) with coordinates based on a laser center position of x = 837 and y = 868.

To determine the ROI based on the laser spot, first, a .fits file is opened in a suitable program, e.g., ImageJ. Subsequently, the image is flipped vertically, and the center coordinates are determined manually (see Fig. S15).

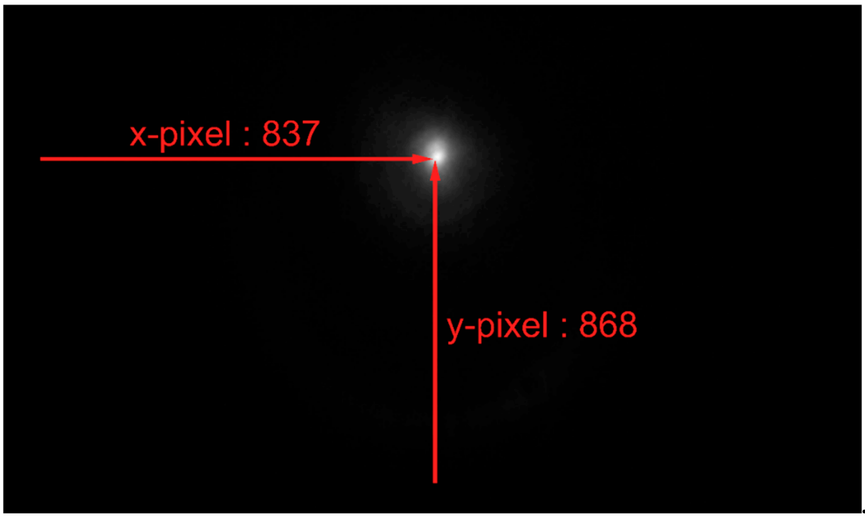

Figure S15. Vertically flipped raw image with highlighted laser center position of 837/868 (x/y).

Based on the determined laser center coordinates, the roi_region_x_y.txt is filled (±25 pixels to create a 50 x50-pixel area) and saved. When the script is launched, the correct file path is entered in the source directory (see Fig. S16), and a suitable name for the output file is chosen, e.g., results.txt (see Fig. S16). The script is executed, and the results are saved in the source directory.

```

##############################################################################
# 3 Data export                                                              #
##############################################################################

fmt = "\t".join(["%s"] + ["%10.3e"] * (results_array.shape[1]-1))
np.savetxt("file_name.txt", results_array, fmt=fmt, header=
"source directory of the raw data: " +"\n"+
"\n"+
"       " +      sourceDir + "\n" +
"\n"+
```

```

##############################################################################
# 1 Working directory:                                                       #
#     a )sets the directory and imports txt files                            #
##############################################################################


sourceDir = "C:\\Users\\martin\\Desktop\\cocaine\\data\\"


if not os.path.isdir(sourceDir):
    raise NotADirectoryError(sourceDir)
```

Figure S16. The path to the raw data with all required input files is entered in the script (top), and the name of the output file (file_name.txt) is defined (bottom).

Table S1**.** Preparation of the Benzoylecgonine-(BEC)-BSA column. The silanization solution contained 1% (v/v) of lab water and 1% (v/v) of diethoxy(3-glycidyloxypropyl)methylsilane (97%) in ethanol. After the affinity coating, the column was stored under 80 % ethanol at 4°C and remained stable for several months.

**Reagent**	**t (min)**	**Flow rate (mL min^{-1})**
Water	10	1
10 (w/v) % KOH + 0.05 (v/v) % Tween20	10	0.5
Water	10	1
1 M HCl	70	0.1
Water	10	1
PBS pH = 7.4	10	1
EtOH (> 99 %)	10	0.5
Silanization solution	7	0.5
Incubation	4 days	0
EtOH (>99 %)	10	0.5
100 mM PBS pH = 7.8	10	0.5
Incubation with BEC-BSA (1 mg/ml)	7 days	0.5
EtOH 50 (v/v) %	10	1
